# Discovery of a Family of Genomic Sequences Which Interact Specifically with the *c-MYC* Promoter to Regulate *c-MYC* Expression

**DOI:** 10.1371/journal.pone.0161588

**Published:** 2016-08-23

**Authors:** Francine Rezzoug, Shelia D. Thomas, Eric C. Rouchka, Donald M. Miller

**Affiliations:** 1 James Graham Brown Cancer Center, Department of Medicine, University of Louisville, Louisville Kentucky, United States of America; 2 Department of Computer Engineering and Computer Science, Speed School of Engineering, University of Louisville, Kentucky, United States of America; Saint Louis University, UNITED STATES

## Abstract

G-quadruplex forming sequences are particularly enriched in the promoter regions of eukaryotic genes, especially of oncogenes. One of the most well studied G-quadruplex forming sequences is located in the nuclease hypersensitive element (NHE) III_1_ of the *c-MYC* promoter region. The oncoprotein c-MYC regulates a large array of genes which play important roles in growth regulation and metabolism. It is dysregulated in >70% of human cancers. The silencer NHEIII_1_ located upstream of the P1 promoter regulates up-to 80% of *c-MYC* transcription and includes a G-quadruplex structure (Pu27) that is required for promoter inhibition. We have identified, for the first time, a family of seventeen G-quadruplex-forming motifs with >90% identity with Pu27, located on different chromosomes throughout the human genome, some found near or within genes involved in stem cell maintenance or neural cell development. Notably, all members of the Pu27 family interact specifically with NHEIII_1_ sequence, in vitro. Crosslinking studies demonstrate that Pu27 oligonucleotide binds specifically to the C-rich strand of the NHEIII_1_ resulting in the G-quadruplex structure stabilization. Pu27 homologous sequences (Pu27-HS) significantly inhibit leukemic cell lines proliferation in culture. Exposure of U937 cells to the Pu27-HS induces cell growth inhibition associated with cell cycle arrest that is most likely due to downregulation of c-MYC expression at the RNA and/or protein levels. Expression of SOX2, another gene containing a Pu27-HS, was affected by Pu27-HS treatment as well. Our data suggest that the oligonucleotides encoding the Pu27 family target complementary DNA sequences in the genome, including those of the *c-MYC* and *SOX2* promoters. This effect is most likely cell type and cell growth condition dependent. The presence of genomic G-quadruplex-forming sequences homologous to Pu27 of c-MYC silencer and the fact that they interact specifically with the parent sequence suggest a common regulatory mechanism for genes whose promoters contain these sequences.

## Introduction

The presence of secondary structure in guanine-rich oligonucleotides was first documented in the late 1980’s [1]. Four adjacent guanines (on one strand or on different strands of DNA) can spontaneously arrange in a square planar structure which is stabilized by Hoogsteen hydrogen bonds called G-tetrads. This structure is further stabilized by monovalent cations at physiological concentrations [1, 2]. G-quadruplex motifs are stable three-dimensional structures that result from stacks of G-tetrads. G-quadruplex forming sequences are highly represented in all living organisms [3, 4]. In the human genome the number of potential G-quadruplex forming sequences has been estimated to be 376,000 [5, 6]. More recently, high resolution sequencing techniques have identified at least 716,000 potential G-quadruplex forming sequences [7]. G-quadruplex-forming sequences were initially identified in the immunoglobulin switch region of the IgG gene [1] and in telomeres [2] where they are highly enriched. G-quadruplex forming sequences are preferentially located near the promoter regions of eukaryotic genes, especially of oncogenes including c-MYC [8, 9], KRAS [10], c-KIT [11] and BCL2 [12]. Several of these sequences, including the c-MYC promoter G-quadruplex-forming sequence, have been shown to be negative regulators of transcription. Interestingly, they are less commonly found in the promoters of tumor suppressor genes [13]. The past 20 years have seen an evolving interest in G-quadruplex structures as targets for cancer therapy primarily due to the putative regulatory role of these structures [14, 15].

One of the most well studied G-quadruplex forming sequences is located in the promoter region of the c-MYC oncogene. The c-MYC gene product is a transcription factor that can activate and/or repress the expression of a large array of genes [16] that are essential for multiple cell functions including proliferation, metabolism, differentiation, adhesion and apoptosis [17–20]. Not surprisingly, c-MYC is required in the transcription factor cocktail for the generation of induced Pluripotent Stem Cells (iPSC) and maintenance of “stemness” along with SOX2, OCT4 and KLF4 [21, 22]. In hematopoietic homeostasis, c-MYC plays an important role in maintaining the balance between proliferation/differentiation and apoptosis of hematopoietic stem cells [23]. Considering its importance in cell function, it is not surprising that deregulation of c-MYC is a key factor in many types of malignancy [24, 25], often associated with increased tendency to metastasis and poor prognosis [26, 27]. Notably c-MYC is abnormally expressed in many aggressive hematologic malignancies including Burkitt lymphomas and multiple myeloma (due to chromosomal translocation [28–30]), acute myeloid leukemia (due to gene amplification [31]) and in others (due to mutations that prolong the protein half-life [32]).

The involvement of c-MYC in all basic cell functions implies the necessity for tight regulation at RNA and protein levels. The mechanisms involved in the transcriptional regulation of *c-MYC* are multiple and complex. One of the major sites of control for human *c-MYC* transcription has been localized in the region -115 base pairs upstream of the P_1_ promoter which has been designated as the nuclease hypersensitive element (NHE) III_1_ This sequence controls >80% of c-MYC expression [8, 33–35]. The NHEIII_1_ sequence contains a high Cytosine (C) to Guanine (G) ratio and has been shown to form secondary DNA structures: the C-rich coding strand forms *i*-tetraplex structures (*i*-motif) and the complementary G-rich non-coding strand forms G-quadruplex structures [8, 9]. NHEIII_1_ regulates *c-MYC* transcription [9, 29, 36] and consequently constitutes a good target for anticancer drugs. The purine-rich strand which forms G-quadruplex structures is part of the transcriptional silencer element, and contains a 27 nucleotide sequence known as Pu27 [9, 37]. Mutations in the NHEIII_1_ element destabilize the G-quadruplex formation and have been reported to result in increased *c-MYC* transcription in vitro [9, 38], implying that transcription is repressed when the Pu27 sequence is in the G-quadruplex form. Consequently, strategies have been devised to stabilize the NHEIII_1_ or G-quadruplex using small molecules to inhibit transcription [39–41]. We have used a synthetic oligonucleotide sequence encoding Pu27 to inhibit leukemic cell growth and have shown that downregulation of c-MYC expression is associated with inhibition of cell proliferation and induced cell death, although no effects were observed on normal hematopoietic cells [42].

In addition to their role in transcription regulation, G-quadruplex structures have been shown to be involved in DNA replication by slowing or stopping the replication machinery complex progression and in meiosis where G-quadruplexes overlap with DNA double-strand break sites [4]. They also appear to play important roles in regulation of translation through 5’UTR mRNA containing G-quadruplex structures (review in [43, 44]). However, the function of such sequences is still poorly understood, especially when one considers the disproportionate number of putative G-quadruplex forming sequences in the genome and the number of genes containing such sequences.

We report here the discovery of seventeen putative G-quadruplex-forming DNA sequences homologous to the parent c-MYC Pu27 genomic (Pu27ge) sequence. These sequences are located on different chromosomes throughout the human genome. We have established that each oligonucleotide sequence of the Pu27 family forms a stable G-quadruplex structure, binds in a sequence specific manner to the NHEIII_1_ of the *c-MYC* promoter and inhibits cell growth of leukemia cell lines. This growth inhibition is accompanied by cell cycle arrest at G1 and S phases and correlates with a decrease in c-MYC expression. Finally we explore the possible role of these sequences by determining their presence in the transcriptome. Our data imply that this family of genomic DNA sequences may be involved in physiologic regulation of c-MYC as well as genes containing such sequences in their promoter regions. In the case of c-MYC regulation, we suggest that this may occur by binding of Pu27 present in the 5’UTR-mRNA transcripts to the complementary sequence NHEIII_1_ in the *c-MYC* promoter in a back-loop mechanism and thereby participating in the transcription machinery in a cell and tissue specific manner.

## Material and Methods

### Oligonucleotides

The sequences for Pu27 and 17 homologous oligonucleotides located in different chromosomes were determined using a BLAT search of Pu27 on the human GRch38 genome assembly [UCSC Genome Browser (http://genome.ucsc.edu)]. The oligonucleotides were purchased from Oligos etc. (Wilsonville, OR) (Table 1). The lyophilized oligonucleotides were reconstituted in sterile nuclease free H_2_O (Millipore, MA) at 500μM and stored at -20°C.

**Table 1:** Sequences of Pu27 and Pu27- homologous G-quadruplex forming oligonucleotides.

Name	Chromosome	5' 3’	Gene	Distance
Pu27-	Chr 8 q24.21	TGGGGAGGGT GGGGAGGGTG GGGAAGG	c-MYC +	0
Pu1-*	Chr 1 p36.31	TGGGAGGTGG GGAGGAGGGT TGGGAAGG	PLEKHG5 -	0
Pu1.2-	Chr 1 p13.3	TGGGGAGGGT GGGGAGGCCG GG	MYBPHL -	0
Pu2+	Chr 2 p11.2	TAGGGAGGGT AGGGAGGGTG GGGAGGG	Antibody Part	50Kb
Pu3-	Chr 3 p22.1	TGGGGAGGGT GGGGAGGGTG GG	MYRIP +	10Kb
Pu3+	Chr 3 q26.33	TGGGGAGGGT GGGGAGGGCG GGG	SOX2 +	0
Pu5-	Chr 5 q35.3	TGGGGAGGGT GGGGAGGGTG GTGAGGGTGG GGAGGGGGAA GG	GRM6 -	0
Pu7+	Chr 7 p22.2	TGGGGAGGGT GGGGAGGGTG GGGAGGG	SDK1 +	0
Pu9-	Chr 9 q21.31	GGGT GGGGAGGGTG GGGAAG	TLE4 +	50Kb
Pu9.2-	Chr9 p21.1	GGGGAGGGT GGGGAGGGGA TGGAA	BC022036 -	0
Pu10.1-	Chr 10 p11.1	GGGAGGGT GGGGAGGGTG GGGAGGG	ZNF37A +	10Kb
Pu10.2-	Chr 10 q11.21	GGGT GGGGAGGGTG GGGAAGG	LINC00841 +	16Kb
Pu11+	Chr 11 p15.1	GGGGAGGAA GGGGAGGGTG GGGAGGG	NAV2 +	0
Pu14+	Chr14 q24.3	GAGGGT GGGGAGGGTG GATGAGGAAGG	SPTLC2 -	0
Pu16+	Chr 16 q12.1	TGGGGAGGGT GGGGAGGGTG G	BRD7 +	60Kb
Pu17+	Chr 17 q25.1	GAGGGT GGGGAGGGTG GGGA	RPL38 +	70Kb
Pu20-	Chr 20 q13.33	GGGGAGGGT GGGGAGGGAG CTGGGGA	CDH4 +	0
PuX+	Chr X p11.4	TGGGGAGGGT GGGGAGAGGC GGGGTGGGGA GGG	TM4SF2 +	0

*the sign indicates the DNA strand on which the sequence or gene is located

### Circular Dichroism (CD) spectrometry

Oligonucleotide samples were diluted at 5μM in TM buffer (50mM TRIS-HCl/2.5mM MgCl_2_, pH7.0), denatured and annealed overnight at room temperature (RT). The concentration was verified by measuring the OD at 260nm in a Diode Spectrometer before the CD measurement on a Jasco J-710 spectropolarimeter in a 1cm Quartz cuvette. Spectra were recorded from 220nm to 340nm at 20°C; four spectra were averaged for each reading. Molar extinction coefficients were calculated using the IDT calculator (IDT.com, oligoAnalyser3.1). CD data were normalized to strand concentration and are expressed in Molar ellipticity [θ] (deg x cm^2^ x decimole^-1^).

### Oligonucleotide structure

Oligonucleotides were 5’-labeled with ^32^P-ATP (3μCu) using T4 PNK and 1x kinase buffer for 20 minutes at 37°C. Unincorporated nucleotides were removed using G-25 Sephadex columns. Samples (4,000 cpm) were then mixed with cold oligonucleotide, boiled for ten minutes and allowed to anneal overnight. An equal volume of 2x glycerol dye was added and run on a 15% native acrylamide gel at 250V for 3h. Bands were visualized by exposing a Kodak phosphor-imager screen and scanned using a Molecular Imager (Pharos FX Plus, Bio-Rad) then to Kodak film for 12h at -80°C.

### Electrophoretic mobility shift assay (EMSA)

PCR product of 134bp covering the NHEIII_1_ of the c-MYC promoter (TS), was incubated with 100,000cpm of ^32^P-labeled Pu27 or Pu-HS in 20mM HEPES (pH 7.9), 25mM KCl, 2mM MgCl_2_, 0.1mM EDTA, 0.2mM DTT, 2mM spermidine and 10% glycerol for 15 min at 37°C. For the competition assay, increasing concentrations of “cold” Pu27 oligonucleotide (1nM to 1μM) were added to each labeled oligonucleotide and incubated for an additional 15 min. After incubation, an equal volume of 2x glycerol dye was added. Complexes (50,000cpm) were resolved by electrophoresis on a 5% non-denaturing polyacrylamide gel, and visualized as described above.

### Cross-linking experiment

The target sequences (TS) double strand (ds), C-rich and G-rich single strand (ss) (IDT, Coralville IA) covering the c-MYC NHEIII_1_ were hybridized with Pu27 or Pu27 containing the cross-linker [3-cyanovinylcarbazole (^CNV^K) kindly provided by Dr. Niles A. Pierce] that was incorporated at the position 1 = PuK1 or position 12 = PuK12 of the Pu27 nucleotide sequence (PuK1 and PuK12 were synthetized by IDT). The cross-linking protocol follows the methodology described by Dr. Pierce [45] with some modifications. The target sequences (1.8μM) were mixed or not with Pu27, PuK1 or PuK12 (3μM) in SSC buffer (150mM NaCl, 15mM trisodium citrate, pH7.0) and annealed for 5min at 95°C in heating block. The samples were then cooled-down at RT for 35min in the dark. Half of each sample was then distributed in a round bottom 96 well plate, placed on ice and irradiated with a UV hand lamp at 365nm for 3min. The samples were then analyzed for crosslinking by: (1). Electrophoresis in denaturing polyacrylamide gel (12% polyacrylamide/8M Urea) using 2.5μl of the samples mixed with 2X glycerol dye before loading on the gel. A 10bp ladder (Invitrogen) was denatured and run in the 1^st^ well for size reference. At the end of the electrophoresis the gel was stained with SYBR® Gold (Molecular Probes) and the bands visualized and photographed in Molecular Imager (Pharos FX Plus, Bio-Rad) and (2). PCR: 1μl of each sample was mixed with primers designed to recognize the 5’-end (Fw) and the 3’-end (Rev) of the 134bp TS (S1 Table) in tubes containing Ready-To-Go PCR beads (GE Healthcare Bio-sciences, Pittsburgh, PA) and run in a one way PCR using one of the primers or in regular PCR with both primers. Electrophoresis using 2% agarose gel was then used to determine the size of the PCR products. Bands were visualized using ethidium bromide and gel photographed with a gel doc (UVP).

### Cell culture

Four leukemia cell lines were investigated: U937 (histiocytic, myeloid lymphoma, AML), Molt-4 (acute-T-lymphoblastic leukemia, ALL), HL-60 (acute promyelocytic leukemia, APL) and Raji (Burkitt’s lymphoma, BL), all obtained from American Type Culture Collection (ATCC, Manassas, VA). Cell lines were maintained in culture in RPMI-1640/10%FBS/penicillin-streptomycin (all from HyClone, Utah) at 37°C/5%CO_2_ in humid atmosphere and were evaluated at exponential growth.

### MTT assay

Cells were seeded at 5x10^3^ cells/well in 96-well flat bottom plates in the presence of different concentrations of oligonucleotides for 24h up to 5 days as specified in the text. Untreated cells were used as control, and media for blank. Cell proliferation was evaluated by using MTT [3-(4,5-Dimethylthiazol-2-yl) 2,5-diphenyltetrazolium Bromide) Sigma-Aldrich, St Louis, MO] [42]. Data are shown as percent of the untreated control for an average of at least 3 experiments+/- Standard deviation.

### Gene and sequences expression by RT-qPCR

U937 were cultured at 1x10^6^cells/5ml in the presence of 10μM Pu27-HS compared to untreated for 72h. At this time point cells were collected and divided in 3 tubes to perform FACS analysis, RNA extraction and protein extraction.

#### For c-MYC or SOX2 expression

Cells for RNA extraction were pelleted, suspended in 5ml TRIzol^®^ reagent (Invitrogen™, NY) and stored at -80°C until samples for 3 separate experiments were collected. RNA extraction was performed following manual instruction and concentration measured on NanoDrop 2000 (NanoDrop, DE). The purified RNA (0.1μg/ sample) was used to generate cDNA with the SuperSript^®^ VILO cDNA synthesis Kit following the instruction manual. To detect the expression of *c-MYC* and *SOX2* primer pairs were designed (sequences in S1 Table), 18S and β-Actin were used as reference genes (all primers from Invitrogen™). SYBR®Green PCR Master Mix (Applied Biosystems®, NY) was used for the RT-qPCR reaction in ViiA™7 (Applied Biosystems®). Gene expression was calculated using C_T_ value and ΔΔC_T_ [46] and expressed as fold change averaged for 3 distinct experiments.

#### For the expression of the different Pu27-HS

RNA was extracted from 1x10^6^ peripheral blood cells from two healthy donors (Innovative Research, Novi MI) and form the 4 Leukemia cell lines previously used in this study as described above. cDNA was generated with 100ng of RNA and evaluated in RTq-PCR. The primers sequences are located in S1 Table, 18S and GAPDH were used as reference genes (all from Invitrogen™) and SYBR®Green PCR Master Mix was used for the RT-qPCR reaction in ViiA™7. Data are represented as comparison of C_T_ values obtained from normal donors and leukemia cells. A C_T_ value above 34 was considered not determined.

### Flow cytometry for cell cycle

U937 were cultured at 1x10^6^ cells/5ml in the presence of 10μM either Pu27-HS compared to untreated cells for 3 days. Cells were collected, washed in PBS and fixed in 70% ethanol and stored at -20°C for at least 2 hrs. Cell suspensions were then washed in PBS and incubated with 5μl 7AAD (eBioscience, Inc., CA) for 20min on ice then analyzed on FACSCalibur (Beckton-Dickinson, NJ). FACS data analyses were performed using the multicycle analysis program from FCS Express4 software (DeNovo Software, CA). Data are expressed as percent of cells in each cell cycle sub-group averaged for 3 separate experiments.

### Western blot

U937 were cultured at 1x10^5^ cells/5ml in the presence of Pu27 or Pu27-HS (10μM) compared to untreated cells for 3 days. Total protein lysates was prepared using M-Per lysis buffer (Pierce Biotechnology, Rockford, IL) containing protease inhibitors (Roche Diagnostics, IN) according to manufacturer instruction and quantified using Coomassie Protein reagent (Thermo Scientific, MA). Equal amounts of protein were run in 4–15% gradient gel (Bio-Rad) for 1h at 70 V, and then transferred to PVDF membrane using an Iblot (Invitrogen™). The membrane was blocked with 5% non-fat dry milk, washed in PBST (PBS/0.05% Tween), hybridized for 1h at RT with anti-c-MYC antibody or anti-Sox2 (both from Santa Cruz Biotechnology, Inc. TX), α-tubulin or β-tubulin (both form Cell Singnaling technology, MA), were used to normalize the protein loading followed by washes in PBST, then incubated 1h at RT with goat anti-mouse antibody. The protein was visualized using femto-chemiluminescence reagent (Thermo Scientific™ Pierce™).

### Statistical analysis

All data are expressed as mean +/- standard deviation (SD) between untreated versus treated and assessed using unpaired two-tailed Student T-test and one-way ANOVA. Data were considered significant for p-value < 0.05.

## Results

### Pu27 family of quadruplex-forming sequences has 18 members

**Fig 1:**
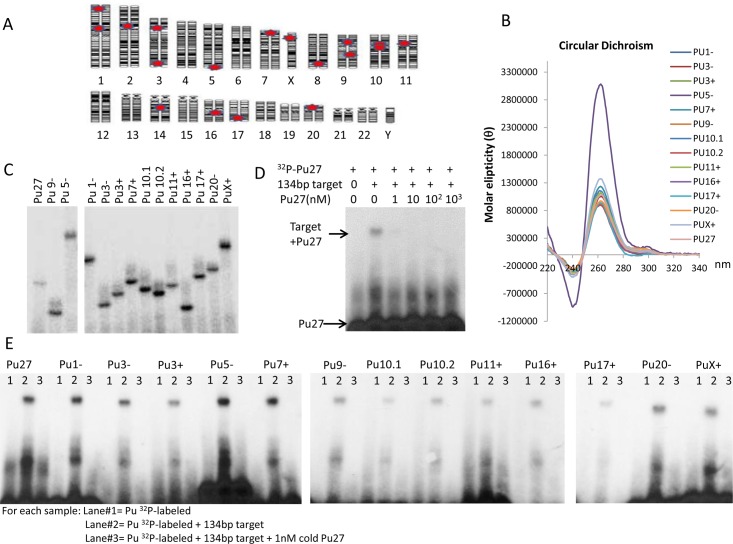
Localization and biophysical analysis of the Pu27 homologous oligonucleotides sequences. (A). Chromosome localization of the Pu27-HS. (B). Circular dichroism spectra for 14 members of the Pu27 family of oligonucleotides showing peaks at 260nm representative of the presence of parallel G-quadruplex. (C). Electromobility shift assays (EMSA), ^32^P-labeled oligonucleotides were run on a polyacrylamide gel that shows unique band for each of the oligonucleotides that migrated.; one representative Phospho imager picture is shown. (D). EMSA for competition assay.^32^P-labeled Pu27 was run in the presence of a 134bp double strand target sequence (TS) containing the NHEIII_1_ of the *c-MYC* promoter +/- different concentrations of cold Pu27; Kodak image is shown. (E). EMSA for competition assay applied to 14 Pu27-HS ^32^P-labeled incubated with 134bp target sequence +/- cold Pu27 at 1nM.

A BLAT sequence similarity search [UCSC Genome Browser (http://genome.ucsc.edu)] with Pu27ge identified 17 homologous sequences (from 88% to 100% homology) localized on different chromosomes (Fig 1A). Most of the Pu27 homologous sequences (hereafter referred to as Pu27-HS) are located within gene transcription regions including the noncoding region of SOX2 /Pu3+, GRM6 /Pu5-, and NAV2 /Pu11+ or on the noncoding strand of the genes (Pu27/c-MYC, Pu20-/CDH4) (Table 1). In several cases the Pu27-HS are located at distance varying from 10Kb to 70Kb from the transcription initiation site of the nearest gene (Table 1). Among the genes containing a Pu27-HS within their transcription regions are genes producing proteins involved in stem cell maintenance such as c-MYC and SOX2 [47] or differentiation TLE4 [48], in brain development and neuron differentiation/function such as NAV2 [49, 50], SDK1 [51], SPTLC2 [52], CDH4 (R-cadherin) [53] and GRM6 [54] (Table 2). Notably, these genes products play a role in embryonic development and in cancer [47, 48, 55, 56].

**Table 2:** Localization of the different Pu27 homologous sequences.

	Gene	Function	DNase I hypersensitive site	Tissue specificity
**Pu27**	In noncoding region between promoter P0 and P1 of *c-MYC*	proliferation, apoptosis, cell cycle and differentiation	yes	no
**Pu1-**	in intron 4 of *PLEKHG5*: Homo sapiens pleckstrin homology domain containing, family G (with RhoGef domain) member 5	activates the nuclear factor kappa B (NFKB1) signaling pathway	yes	no
**Pu1.2-**	UTR of *MYBPHL*: Homo sapiens myosin binding protein H-like	No data available	yes	ND
**Pu2+**	~3.5Kb from *IGKV3D-7*: immunoglobulin kappa variable 3D-7	immunoglobulin	no	yes
**Pu3-**	Opposite strand in non-coding area near *MYRIP*: Homo sapiens myosin VIIA and Rab interacting protein.	melanosome transport and link melanosome-bound RAB27A and MYO5A and MYO7A	yes	yes
**Pu3+**	in *SOX2*: Homo sapiens SRY (sex determining region Y)-box 2	regulation of embryonic development is required for stem-cell maintenance	yes	yes
**Pu5-**	in intron 5 of *GRM6*: Homo sapiens glutamate receptor, metabotropic 6.	role in neurotransmission	yes	yes
**Pu7+**	In intron 23 of *SDK1*: Homo sapiens sidekick cell adhesion molecule 1.	guides axonal terminals to specific synapses in developing neurons	yes	yes
**Pu9-**	In a non–protein coding region of the DNA. The nearest transcribed element is a long non coding RNA *LINC01507*: Homo sapiens long intergenic non-protein coding RNA 1507. The nearest coding gene is *TLE4* (~50Kb) Homo sapiens transducin-like enhancer of split 4.	ND	yes	ND
**Pu10.1-**	on the opposite strand at ~7Kb from gene *ZNF37A*: Homo sapiens zinc finger protein 37A (Zinc finger protein KOX21)	ND	yes	
**Pu10.2-**	On the opposite strand at ~16Kb from *LINC00841*: Homo sapiens long intergenic non-protein coding RNA 841, long non-coding RNA	ND	yes	ND
**Pu11+**	In intron 1 of *NAV2*: Homo sapiens neuron navigator 2.	role in neuron growth and migration, neuronal development, specifically in the development of sensory organs	yes	yes
**Pu14+**	On the opposite strand in intron 2 of *SPTLC2*: Homo sapiens serine palmitoyltransferase, long chain base subunit 2	sphingolipid biosynthesis	yes	yes
**Pu16+**	Closest gene at ~60Kb on the opposite strand of *BRD7*: Homo sapiens bromodomain containing 7.	BRD7 interacts with p53 and is required for p53-dependent oncogene-induced senescence which prevents tumor growth	no	no
**Pu17+**	In intron 1 of RP11-101O21.1, and at ~68Kb of *RPL38*: Homo sapiens ribosomal protein L38.	Ribosomal protein that is a component of the 60S subunit.	no	no
**Pu20-**	On the opposite strand in intron 2 of *CDH4*: Homo sapiens cadherin 4, type 1, R-cadherin (retinal). This gene encodes a protein member the cadherin superfamily.	Calcium-dependent cell-cell adhesion glycoproteins play an important role during brain segmentation and neuronal outgrowth.	no	yes
**PuX+**	In intron 3 of RP5-972B16.2: Human Gene RP5-972B16.2. The protein encoded by this gene is a member of the transmembrane 4 superfamily, also known as the tetraspanin family.	Cell surface glycoprotein control of neurite outgrowth and complex with integrins.	no	yes

Extracted form NCBI gene

ND: not determined

**Fig 2:**
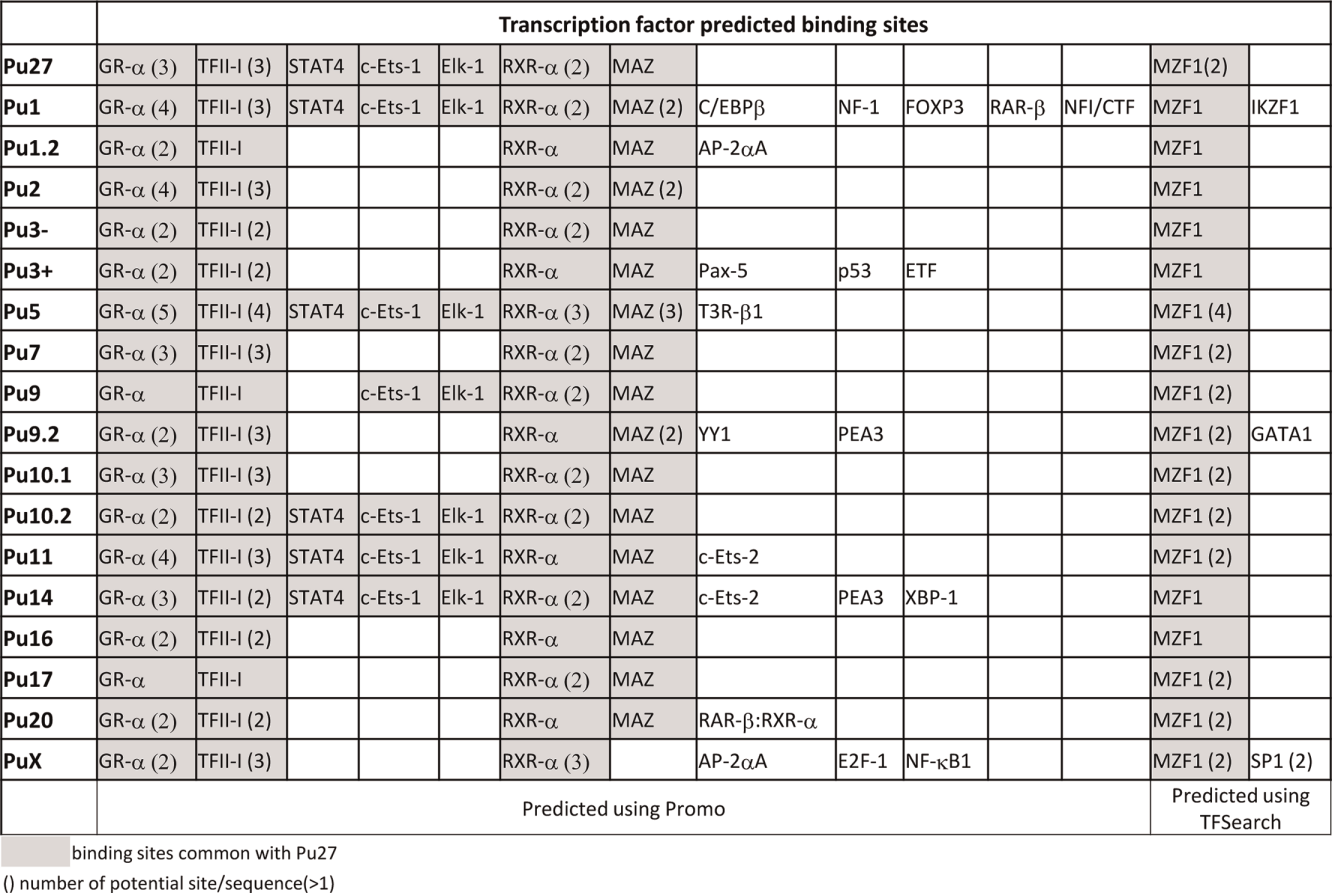
Table of predicted transcription factors binding sites in the Pu27 homologous sequences.

The low complexity and repetitive pattern of the Pu27-HS suggests the presence of binding sites for transcription factors (TF). In fact, it has been shown that the P0-P1 region of *c-MYC* contains binding sites for Nucleolin, SP1 [57–60] and 4 putative binding sites for Myeloid Zinc Finger 1 (MZF1) [61]. Therefore we performed a search for the presence of possible TF binding sites within the Pu27-HS sequences using readily available bio-informatics websites [(http://alggen.lsi.upc.es/cgi-bin/promov3/promo/promoinit.cgi?dirDB=TF_8.3), (http://www.cbrc.jp/research/db/TFSEARCH.html)]. Common binding sites for GR-α, TFII-I, RXR-α and MZF1 (with 1 to 4 binding sites) were identified within all members of the Pu27 family. Putative binding sites for STAT4, c-ETS1 and ELK-1 were also found in most of the Pu27-HS, while SP1, IKZF1, GATA1 or P53 were found in 1 or 2 sequences only (Fig 2).

### All of the Pu27 homologous sequences form G-quadruplex

Although the Pu27-HS are of different lengths, they all contain a common core of four consecutive runs of three to four guanines suggesting the capability to form G-quadruplex structures. Therefore oligonucleotides encoding each Pu27-HS were analyzed by Circular Dichroism spectrometry (CD). All of the Pu27 family members formed an intramolecular parallel G-quadruplex evidenced by the presence of a peak of Molecular Ellipticity (θ) at 260-262nm (Fig 1B and S1A Fig). The higher amplitude obtained with Pu5 can be an indicator of the number of G-tetrad stacks as Pu5 contains 7 runs of 3–4 guanines compared to Pu27 (5 runs). However, it could also be due to the formation of “G-wire” structures which may be observed with long sequences. In order to evaluate the presence of secondary structures, Pu27-HS were subjected to an electromobility shift assay (EMSA). Most Pu27-HS displayed no secondary structures (hair pin, duplex or wires) as shown by the presence of unique bands. However, Pu9 demonstrated a faint band of larger molecular weight which may be due to dimer formation (Fig 1C). The presence of a unique band for Pu5 confirms that the CD amplitude is most likely due to the number of G-tetrad stacks, ruling out the possibility of wire formation.

### All Pu27 homologous sequences bind specifically to the NHEIII_1_ target sequence

Next we evaluated whether Pu27 binds to its target sequence (TS) in the *c-MYC* promoter region [37, 62]. The binding target is a double stranded (ds) DNA fragment of 134bp derived from the *c-MYC* promoter region containing the proximal NHEIII_1_ sequence. EMSA experiments show high affinity binding of Pu27 to the TS (Fig 1D and S1B Fig: lane 2), that was competed by adding increasing concentrations of unlabeled Pu27. This competition assay shows a dose-dependent displacement of the ^32^P-labeled Pu27/target band (Fig 1D lanes 3–6) with 1nM Pu27 being the minimal concentration required to fully compete with ^32^P-Pu27. Importantly, other quadruplex-forming oligonucleotides such as KRAS did not compete with Pu27 for binding to the TS (S1C Fig). These data strongly suggest that Pu27 binds specifically to NHEIII_1_, stabilizing the genomic G-quadruplex structure thus, inhibiting *c-MYC* transcription.

The gel shift assay was also used to characterize the binding of the Pu27-HS to the NHEIII_1_ sequence and demonstrate that all of the Pu27-HS oligonucleotides bind to the TS (Fig 1E and S1B Fig: lane 2) and were displaced by unlabeled-Pu27 (Fig 1E and S1B Fig: lane 3). EMSA control with other G-quadruplex sequences such as ^32^P-AS1411 [63] or with ^32^P-KRAS [10] showed no binding to the TS (S1D Fig). Taken together these data demonstrate the specificity of the Pu27-HS binding to the NHEIII_1_ parent sequence and suggest that the binding occurs at the same site of *c-MYC* promoter region.

### Pu27-homologous sequences inhibit leukemic cells proliferation

**Fig 3:**
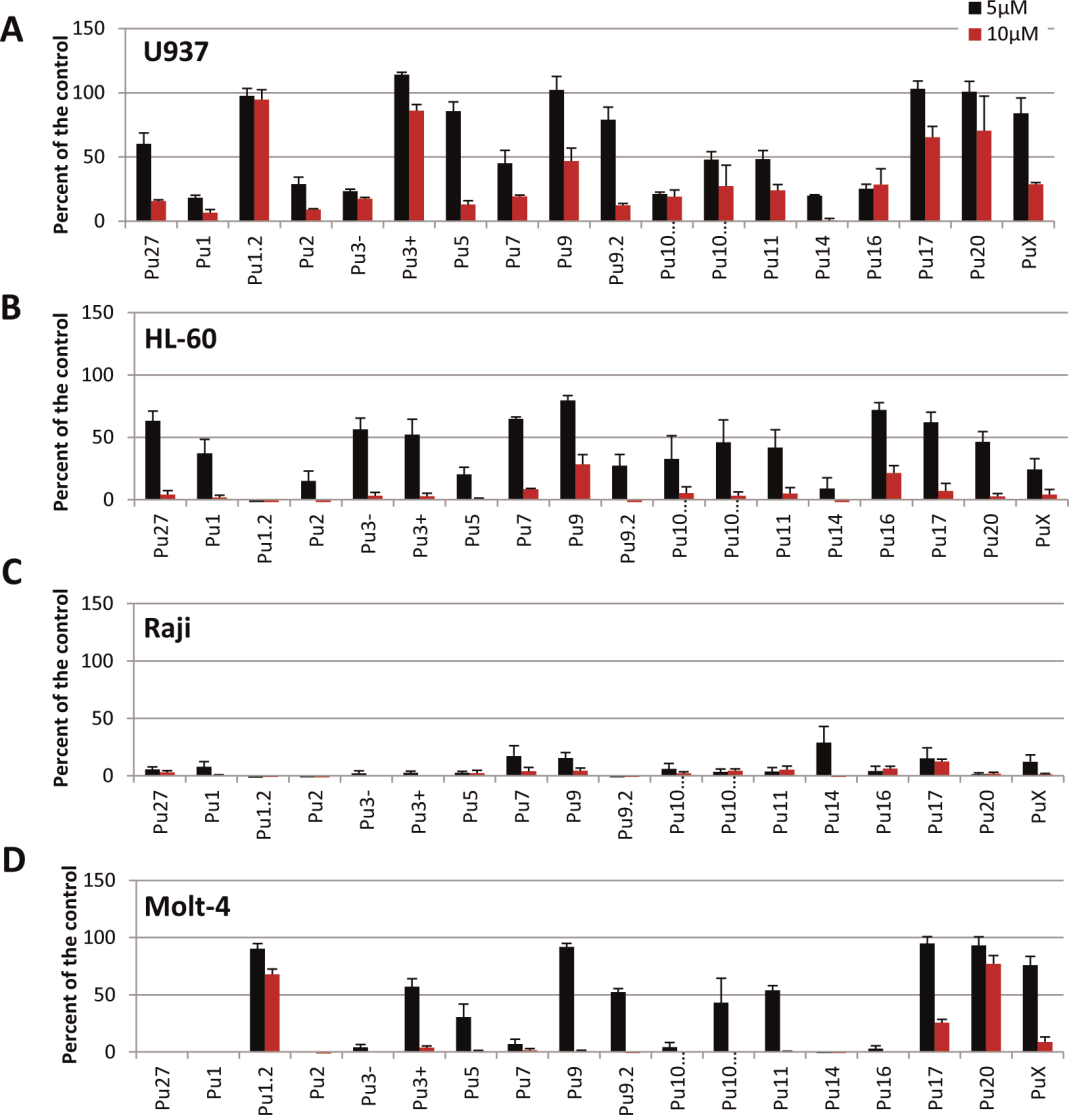
Growth inhibition assay for four different leukemia cell lines exposed to the Pu27-HS. **(**A) U937, (B) HL-60, (C) Raji, and (D) Mol-4: 5000cells/well were exposed to 5 or 10μM of Pu27 homologous oligonucleotides sequences for 5 days then evaluate for growth inhibition in MTT assay. Data report the average+ SD of at least 3 different experiments (*p<0.05).

We have previously shown that Pu27 downregulates c-MYC expression and inhibits the growth of leukemia cell lines [42]. We therefore assessed the ability of the Pu27-HS oligonucleotides to inhibit the growth of four leukemia cell lines at 5μM and 10μM in 5 day cultures using MTT assay. The effect on cell growth was dose and cell line dependent and the four cell lines investigated in this study show significant inhibition of cell proliferation. Overall, the U937 cell line was less sensitive to Pu27-HS < HL-60 < Molt-4 < Raji (Fig 3). Notably, all the Pu27-HS oligonucleotides restricted leukemia cell growth, in some cases more effectively than Pu27, such as Pu1, Pu2 or Pu3-. Interestingly, the relative sensitivity of each cell line appears to be different for each Pu27 family member. For instance, U937 cells demonstrate only 30% or less inhibition in the presence of Pu1.2, Pu3+, Pu9, Pu17 or Pu20 (Fig 3A), while HL-60 seems to be less responsive to Pu9 and Pu16 (Fig 3B) and Molt-4 to Pu1.2, Pu17 or Pu20 (Fig 3D). All Pu27-HS reduce cell growth by 90% and greater for Raji (Fig 3C). The cell sensitivity to Pu27-HS may depend on (de)regulation (mutation, translocation…) of *c-MYC* or of other genes containing these sequences and on the presence of NHEIII_1_ in the promoter region. Furthermore, the degree of sensitivity could relate to the extent to which proliferation of each cell type is dependent on c-MYC overexpression. For all cell lines, the most potent growth inhibiting sequences were Pu27, Pu1, Pu2, Pu3-, Pu5, Pu7, Pu10.1, Pu10.2 and Pu11.

**Fig 4:**
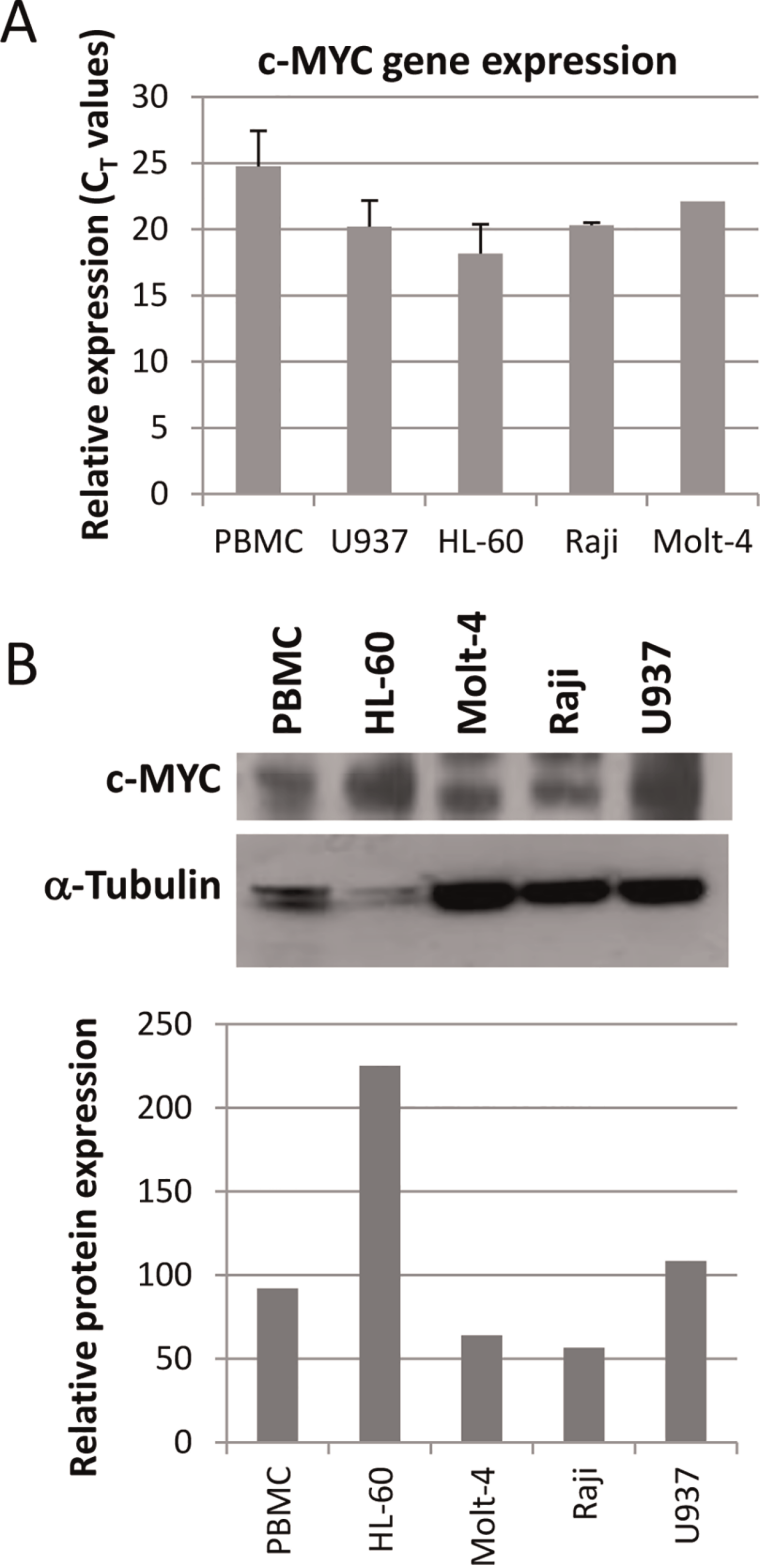
Expression of c-MYC in four leukemia cell lines and 2 healthy donors PBMC. **(**A). Bar graph representing RT-qPCR for relative expression (C_T_ values) of *c-MYC* in 4 leukemia cell lines and PBMC. (B) Western blot for c-MYC protein expression. (C). Bar graph for the quantification of protein normalized to α-tubulin.

The AML cell line U937 was used to further investigate the effect of the Pu27–HS in cell culture. This cell line overexpresses c-MYC relative to normal peripheral blood mononuclear cells control (Fig 4A and 4B) most likely due to the trisomy of chromosome 8 [64] but not from translocation therefore c-MYC expression should remain under the control of the NHEIII_1_.

### Pu27–HS control c-MYC expression

**Fig 5:**
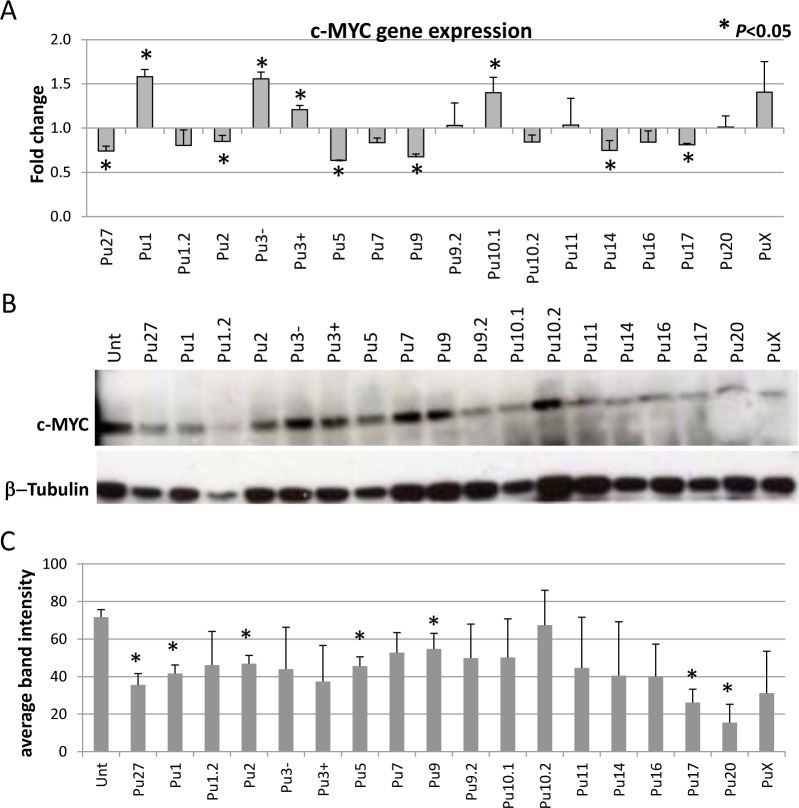
Effect of Pu27-HS on c-MYC expression in U937 after 3 days exposure. (A). Evaluation of c-MYC gene expression by RT-qPCR after 72h exposure to 10μM Pu27-HS oligonucleotides. Data represent the average in fold change compared to untreated +/- SD of 3 independent experiments (*p<0.05). (B). Evaluation of c-MYC protein expression by Western Blot, representative blot is shown. (C). Quantification of c-MYC normalized to β-Tubulin, bar graph shows the average of intensity of the band for 3 separate experiments (*p<0.05).

One possible mechanism by which the Pu27-HS oligonucleotides inhibit leukemic cell proliferation is by downregulating c-MYC expression as previously shown for Pu27 [42]. Therefore, U937 cells were exposed to either of the Pu27-HS at 10μM for 72h and *c-MYC* transcription was evaluated by RT-qPCR analysis of total RNA. There was a significant downregulation in *c-MYC* expression in the cells treated with Pu27, as expected, and with five of the Pu27-HS (Pu2, Pu5, Pu9, Pu14 and Pu17) (Fig 5A), four of Pu27-HS downregulated *c-MYC* expression less efficiently (Pu1.2, Pu7, Pu10.2, Pu16). However, *c-MYC* was significantly upregulated in U937 exposed to four of the Pu27-HS: Pu1, Pu3-, Pu3+ and Pu10.1 (Fig 5A), four Pu27-HS did not affect *c-MYC* expression at that time point. The difference observed at the transcription level could be due to difference in specificity -as we expect Pu27 will have higher specificity for its target- or difference in regulation (upregulation instead of downregulation) for these particular Pu27-HS (i.e. Pu1, Pu3-,Pu3+ or Pu10.1). Given the fact that all Pu27-HS can bind to the DNA sequence containing Pu27 target and that all sequences induce growth inhibition at 5 days we wanted to verify the effects of the Pu27-HS on c-MYC protein expression using western blotting assay. There was a marked reduction of c-MYC in all treatment groups compared to untreated control even in cells where there was no change or upregulation of c-MYC transcription (Fig 5B and 5C) suggesting delay or anomalies in the translation.

### Pu27 binds to C-rich strand in the NHEIII_1_ of *c-MYC* promoter

**Fig 6:**
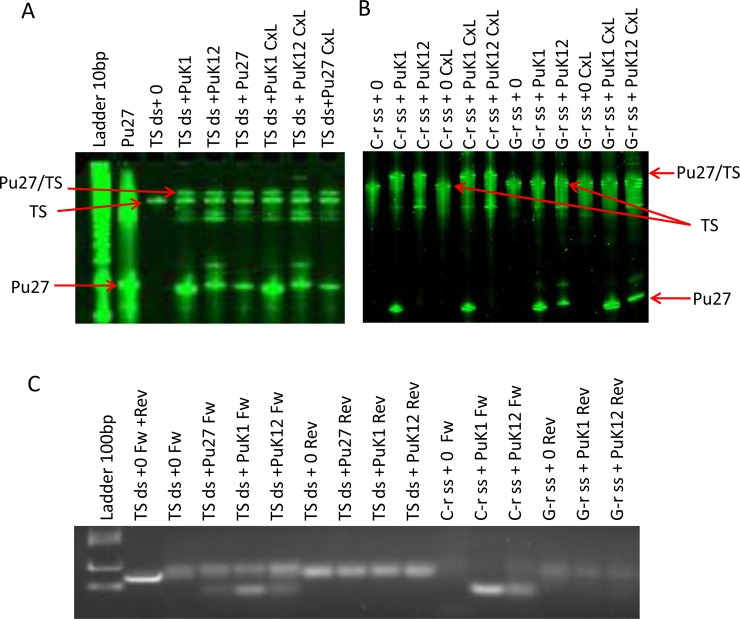
Pu27 binds to the C-rich strand in the NHEIII_1_ of c-MYC promoter. (A). Polyacrylamide denaturing gel electrophoresis of product of hybridization of Pu27 and Pu27 containing the crosslinker 3-cyanovinylcarbazole (^CNV^K): PuK1 and PuK12 with the 134bp double strand target sequences (TS ds) before and after crosslinking (CxL). (B). Same experiment for hybridization of PuK1 and PuK12 with the C-rich (C-r ss) or G-rich (G-r ss) single strands of the TS. Two representative gels are shown; the binding of Pu27 is evidence by the presence of an extra band (Pu27/TS) of higher molecular weight. (C). Agarose gel for the size of PCR product of the crosslinking samples. One way PCR was realized using “Fw” primer to identify the C-rich strand and “Rev” primer to identify the G-rich stand. The binding of Pu27 to the TS was confirmed by the presence of a smaller band in the C-rich strand.

To further characterize the binding of Pu27 to its target sequence and determine the mechanism by which this binding could alter *c-MYC* transcription, we took advantage of a UV activated cross-linker to covalently bind the DNA target sequence and the Pu27 encoding oligonucleotide [45]. The cross-linker [3-cyanovinylcarbazole (^CNV^K = K)] is a photoactive nucleoside analogue originally described by Yoshimura et.al. [65, 66], that was incorporated in Pu27 sequence (at the position 1 = PuK1 or position 12 = PuK12). Pu27, PuK1 and PuK12 were hybridized to the target sequence double stranded (TS-ds) previously used in EMSA assay for the binding study, or with the C-rich and G-rich single strands of the same sequence (C-r ss, G-r ss), then exposed to UV light for crosslinking in order to create a covalent bond (CxL). The presence of binding was confirmed by electrophoresis that revealed a second band of higher molecular weight above the migration band of the TS-ds (Fig 6A) and of the C-rich ss TS but not with G-rich ss TS (Fig 6B). As expected from the EMSA experiment, a binding band was also observed with Pu27 without ^CNV^K. Since the crosslink with ^CVN^K is stable even at high temperature [66] we were able to verify the binding using one way PCR with primers designed to recognize each strand of the double strand target. Fig 6C shows an agarose gel run with the PCR products, “Fw” is for the primer reading the C-rich strand and “Rev” for the primer reading the G-rich. Smaller products, indicating Pu27 binding on the strand, were present in TS-ds/PuK1, TS-ds/PuK12 and even in TS-ds/Pu27 (although with less product most likely due to loss of the binding in the absence of the crosslinker) when PCR was performed with the Fw primers reflecting binding to the C-rich strand. This was confirmed with the PCR products derived from the cross-linking experiment of the single stranded targets (C-rich and G-rich) with PuK1 and PuK12 that show small PCR products only for PuK1 and PuK12 hybridization with the C-rich strand. Taken together these data suggest that the oligonucleotide encoding the G-rich Pu27 binds by Watson-and Crick binding to the C-rich strand which is the transcribed strand of the c-MYC gene. This result also suggests that all the oligonucleotide sequence members of the Pu27-HS bind in this manner as well. In addition, this result raises the possibility that Pu27 and/or the Pu27-HS bind to the 5’UTR mRNA that will have resulted from the transcription initiation at the P0 promoter and therefore containing the target sequence.

### Pu27-HS are specific of the nucleotide sequences in the DNA

**Fig 7:**
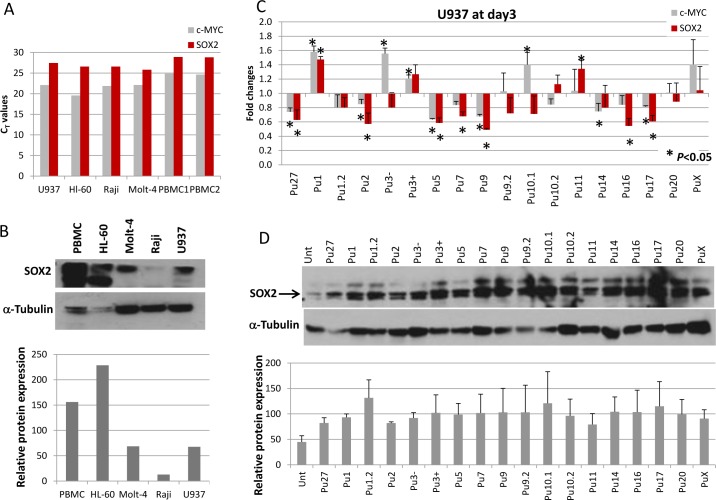
Effect of Pu27-HS on SOX2 expression in U937 after 3 days exposure. (A). Bar graph representing RT-qPCR (C_T_ values) for *SOX2* (red bars) compared to *c-MYC* (gray bars) expression in 4 leukemia cell lines and PBMC from 2 donors. (B) Western blot for SOX2 protein expression in 4 leukemia cell lines and PBMC. Bar graph for the quantification of SOX2 protein normalized to α-tubulin. (C). Evaluation of *SOX2* expression compared to c-MYC after 72h exposure to 10μM Pu27-HS. Data represent the average of fold change for treated compared to untreated +/- SD of 3 independent experiments (*p<0.05). (D). Evaluation of SOX2 protein expression in U937 by Western Blot, representative blot is shown. Quantification of SOX2 normalized to α-Tubulin, bar graph shows the average of intensity of the bands for 2 separate experiments.

Since we have demonstrated that the Pu27-HS are able to modulate c-MYC expression by binding to the target sequence in the *c-MYC* promoter NHEIII_1_, it is conceivable that other genes containing these sequences could be regulated in the same manner. Among the genes containing such sequences, *SOX2* is the most likely to be expressed in the hematopoietic progenitor cells present in leukemia cell lines. The *SOX2* promoter contains a G-quadruplex forming sequence of the Pu27 family: Pu3+. SOX2 is a transcription factor present in stem cells involved in the maintenance of stem cell self-renewal and is also part of the transcription cocktail along with c-MYC for the generation and maintenance of iPSC. Furthermore, SOX2 and c-MYC are present at the same transcription sites of a large variety of genes [67]. We verified the expression of SOX2 in the 4 leukemia cell lines investigated in this study and in PBMC from 2 healthy donors. The *SOX2* gene was indeed expressed in all cells leukemia and normal PBMC (Fig 7A). Similarly the expression of SOX2 protein was confirmed by western blotting in the four leukemia cell lines and PBMC (Fig 7B). However, the protein expression was lower in U937, Molt-4 and especially in Raji compared to HL-60 or to the control PBMC and did not correlate with *SOX2* gene expression.

We then investigated the expression of SOX2 in U937 cells exposed to Pu27-HS for 3 days in the same conditions described for c-MYC expression. The RT-qPCR data revealed that *SOX2* transcription is modulated in the same manner as *c-MYC* for at least for 9 out of 18 Pu27-HS (Fig 7C). For Pu3-, Pu10.1 and Pu14 exposed cells there was no effect on *SOX2* expression while *c-MYC* was upregulated or downregulated, and for Pu11 and Pu16 exposed cells there was no effect on *c-MYC* expression but significant *SOX2* upregulation for Pu11 and downregulation for Pu16 (Fig 7C). Some of the sequences did not affect *SOX2* or *c-MYC* expression such as Pu1.2, Pu9.2, Pu20 and PuX. All together this data suggest that each Pu27-HS provided similar regulation for the genes containing such sequence, however the mechanism of action can be different i.e. Pu27 downregulates while Pu1 or Pu3+ upregulate gene expression. As for c-MYC the gene and protein expression did not always match, we investigated the effect of the Pu27-HS on SOX2 protein expression by western blotting. The SOX2 protein expression was upregulated in all treatments compared to untreated (Fig 7D) suggesting a possible post-transcriptional regulation by some of the Pu27-HS.

### Pu27 homologous sequences induce cell cycle arrest in the G1 and S phases

**Fig 8:**
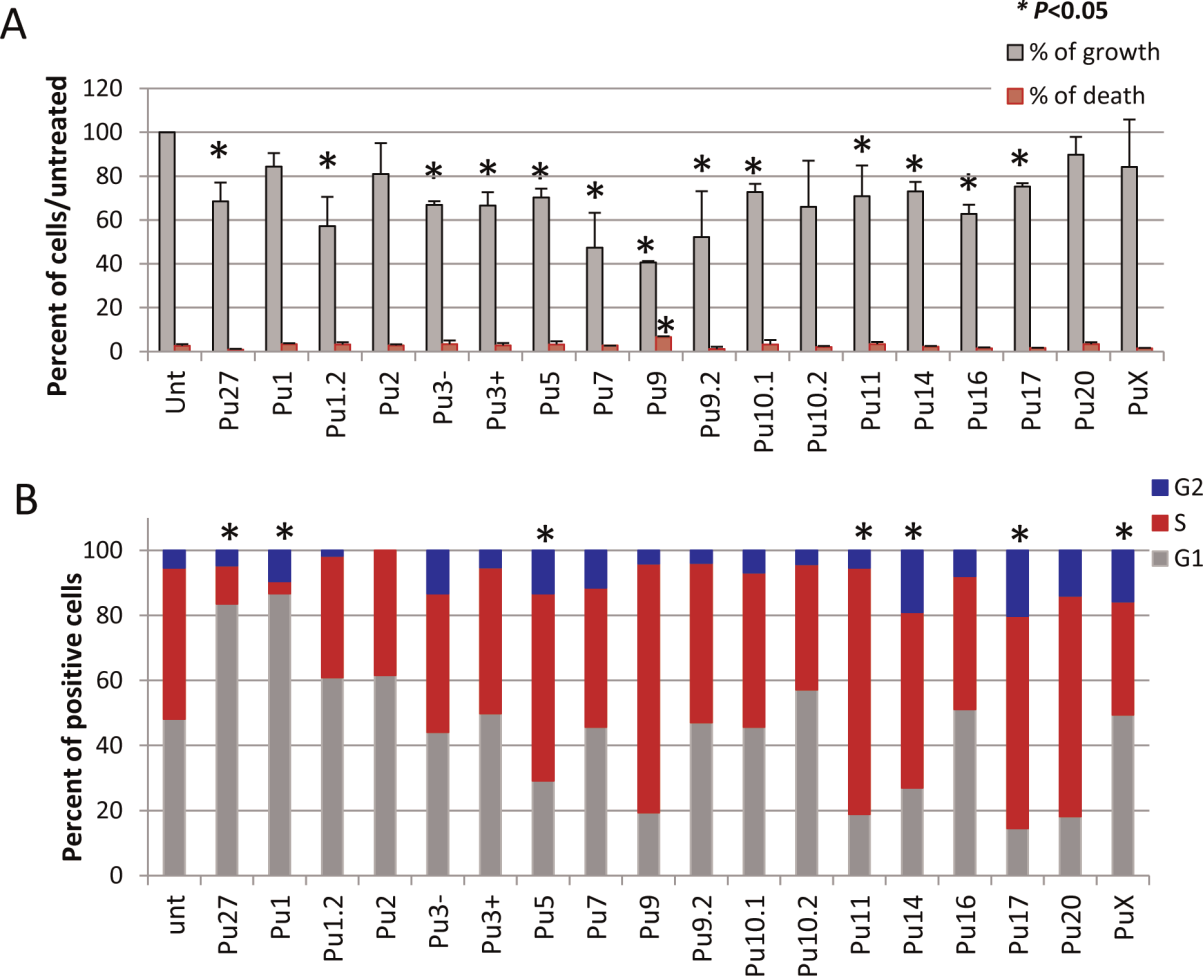
Effect of Pu27-HS on cell cycle progression in U937 after 3 days exposure. (A). Evaluation of percent of cell growth (gray) and percentage of cell death (red) by Trypan Blue after 3 days exposure to 10μM Pu27-HS. (B). Bar graph representing the percentage of cells in G1 (gray), S (red) and G2 (blue) phases of the cell cycle. Averages for 3 independent experiments are shown, *p<0.05.

It is likely that the inhibition of cell proliferation observed in the leukemic cells after 5 days of Pu27 treatment is due to the decrease in c-MYC expression. As c-MYC is involved at different levels in regulating cell proliferation we decided to investigate the cell cycle status of the U937 cell line during exposure to Pu27-HS. U937 cells were grown in the presence of 10μM of the Pu27-HS for 3 days, at which time cells were collected and counted in the exclusion dye Trypan blue to evaluate cell growth and viability. As shown in Fig 8A, by day three cell growth was already significantly reduced in U937 exposed to most of the Pu27-HS compared to untreated; however, there was no significant change in cell viability compared to the control untreated (Fig 8A red bars) except for Pu9 exposed cells where a small but consistent increase in cell death was observed.

While c-MYC is expressed at low levels in quiescent cells, it is induced upon cell division and constitutively expressed throughout the cell cycle [68]. c-MYC is involved at different stages of the cell cycle progression. In addition, overexpression of c-MYC has been suggested to shorten the G1-[69] and the S-phases [70] of the cell cycle in doing so increasing cell division rate. We have previously shown that Pu27 induces cell cycle arrest in G1-phase most likely due to c-MYC downregulation. The analysis of the cell cycle status in U937 exposed to the Pu27-HS confirmed our previous observation of G1 arrest for Pu27 (Fig 8B). In addition to Pu27, cell cycle arrest at the G1-phase was induced by Pu1, Pu1.2, Pu2 and Pu10.2 which would be consistent with c-MYC involvement in the G1/S phase transition [68] suggesting that these Pu27-HS may affect c-MYC and its downstream target in the same manner. In contrast Pu5, Pu9, Pu11, Pu14, Pu17 and Pu20 induced a cell cycle arrest at the S-phase reflecting the role of c-MYC at different check points of the cell cycle. Nevertheless, the accumulation of cells at either G1- or S-phase of the cell cycle will reduce the relative number of dividing cell and eventually result in growth inhibition.

### Some of the Pu27 homologous sequences are present in the transcriptome

**Fig 9:**
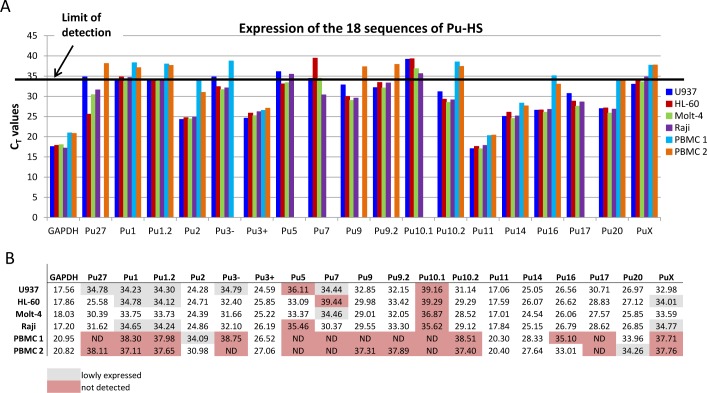
Expression of the 18 Pu27-HS in leukemia cell lines and in healthy donor PBMC. (A). Bar graph for the C_T_ values of RT-qPCR realized with total RNA collected form 4 leukemia cell lines and 2 healthy donor PBMC. (B). Table showing the C_T_ values for expression (white), lowly expressed (gray) and not determined (pink).

As shown in Table 1 most of the Pu27 homologous sequences are located within the untranslated region (UTR) of the mRNA for some genes (c-MYC, SOX2.), or in/or near long non-coding RNA, however some sequences are not located in the vicinity of a gene. In an attempt to understand the role of these sequences in cell function, we investigated whether the Pu27-HS are expressed in the transcriptome of normal and leukemic cells. The total RNA was collected from each cell line at optimal growth and converted to cDNA as was the RNA collected form 2 healthy donors’ peripheral blood mononuclear cells (PBMC). The transcription level of each of the Pu27-HS was evaluated by RT-qPCR assay using primer pairs designed to encompass each Pu27-HS on the same strand. The threshold cycle (C_T_) was considered significant for a number of cycles below 34 for highly expressed and C_T_ values between 34 and 35 for lowly expressed and not detected for C_T_ values above 35. The data show that Pu3+, Pu11 and Pu14 were highly expressed in all four cell lines and PBMC indicating their presence in the untranslated mRNA- while Pu2, Pu16 and Pu20 were expressed in leukemic cells and less in PBMC (Fig 9A and 9B). Some of the Pu27-HS transcripts were present in the leukemia cells but not in the PBMC and some were lowly or not expressed in any of the cells investigated within the scope of this study. The difference in expression of the Pu27-HS between healthy donors PBMC and Leukemia cell lines could reveal a role for these sequences in this malignancy. Notably, Pu27 was lowly transcribed in U937 compared to Molt-4, Raji or HL-60 which seems to express the most correlating Pu27 with c-MYC expression for this cell line. As Pu27 is present in the untranslated region (UTR) of the mRNA, the low level of Pu27 expression in U937 could indicate a low level of UTR-mRNA. The fact that the expression of Pu27 in U937 did not correlate with the expression of c-MYC suggests a truncated or absent 5’UTR-mRNA for this cell line and could reflect the role of such RNA in the gene transcription. Furthermore, the fact that these sequences are expressed at relatively high levels will be consistent with their regulatory role in gene expression.

## Discussion

The estimated number of G-quadruplex forming motifs has increased with the decoding of the human genome [7, 71]. Thus, it is not surprising that some G-quadruplex-forming sequences may be found in multiple copies, as is the case for the G-quadruplex-forming sequences within telomeres or centromeres of the chromosomes, reflecting the fact that they share the same function. However, G-quadruplex-forming sequences contained in regulatory regions of eukaryotic promoters have generally been thought to be unique. Unexpectedly, our search for localization of Pu27 revealed 17 putative G-quadruplex forming sequences with a high degree of homology to the sequence of NHEIII_1_ in the *c-MYC* promoter. These DNA sequences, homologous to Pu27, are located in different chromosomes and often are positioned in or near gene transcription sites.

The oligonucleotides encoding all the Pu27-HS were confirmed to form G-quadruplex and to bind specifically to the double stranded Pu27 parent sequence in NHEIII_1_ of *c-MYC* promoter. Furthermore, we have previously shown that Pu27 inhibits cell growth and downregulates *c-MYC* transcription in leukemic cell lines [42]. We show here that all of the Pu27 family members inhibit cell proliferation as much as, and in some cases even more than Pu27 in four different leukemia cell lines presumably through c-MYC downregulation. The fact that all the Pu27-HS share the Pu27 structure and bind in a sequence-specific manner to the *c-MYC* promoter silencer raises the possibility of a complex regulatory system for c-MYC (and perhaps for the other genes containing such sequences) that may involve intra-chromosomal and inter-chromosomal interactions. Such interactions have been reported for enhancers localized at sites distant from the gene promoter such as in the β-globin cluster gene [72] and even, on other chromosomes (T-helper2 cytokines genes [73]). There is evidence supporting long range control (LRC) of *c-MYC* transcription, as enhancers have been found as far as 335Kb from the *c-MYC* promoter [74, 75] and binding sites for CTCF (CCCTC-binding factor) [76] and TCF-4 are present within the promoter region [77, 78]. At least one binding site for CTCF is located within the complementary strand (C-rich) containing NHEIII_1_ further suggesting a role in LCR for this silencing element. Another possibility is that one or more of these Pu27-HS may be transcribed into a noncoding transcript which will interact specifically with the parent Pu27 target sequence. Such an interaction if it occurs would be expected to stabilize the G-quadruplex structure keeping the transcription in off position and would be sequence and gene specific. In fact, we found that most of the sequences of the Pu27 family are present in the transcriptome. The relative expression of such sequences seems to be proportional to their presence in the UTR of transcribed genes such as SOX2, NAV2 or SPTLC2 and would likely be cell/tissue specific. It is thus possible that in normal cell function the G-quadruplex contained in the newly transcribed mRNA regulates further transcription in a DNA-RNA back-loop binding as it has been demonstrated for c-MYC 5’UTR mRNA [79]. The low representation of Pu27 in the transcriptome of U937 could be a characteristic of this particular cell line and may reflect the lack of control for c-MYC transcription by the G-quadruplex structure present in the 5’UTR-mRNA.

**Fig 10:**
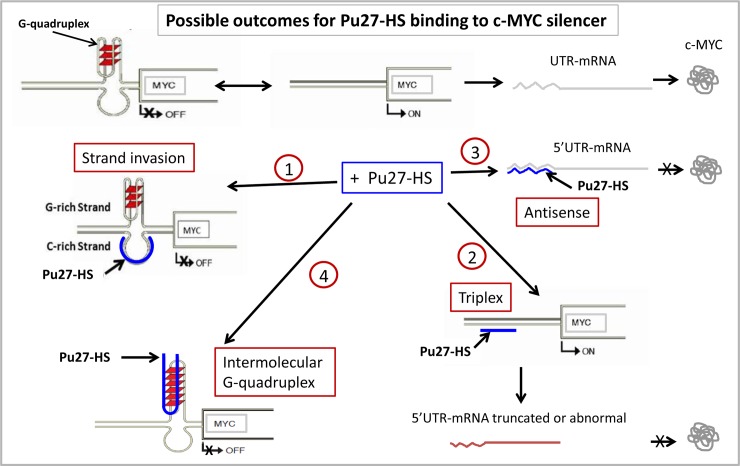
Schematic of mechanisms of action for the Pu27-HS to inhibit c-MYC expression in U937. The silencer NHEIII_1_ in the c-MYC promoter is in equilibrium between the OFF position when it forms G-quadruplex structure or ON when in duplex resulting in mRNA and protein production. In the presence of Pu27-HS (blue) the protein can be inhibited after several possibilities of binding: 1) Pu27-HS bind to the C-rich strand and stabilize the G-quadruplex and inhibit transcription. 2) Pu27-HS bind to C-rich duplex and induce error in the mRNA and inhibit translation. 3) Pu27-HS bind to the C-rich complementary sequence in the 5’UTRmRNA and inhibit translation. 4) Pu27-HS bind to the G-rich, stabilize the G-quadruplex and inhibit transcription.

The fact that most of the Pu27-HS are located within gene transcription sites also suggests a potential role in the regulation of these genes analogous to that of Pu27/NHEIII_1_ on *c-MYC* regulation and therefore they may define concordant pathways for gene regulation. This also predicts cell specificity for these sequences. In this work, the comparison of the effect of the different Pu27-HS on the expression of *c-MYC* and *SOX2* revealed that the majority of these G-quadruplex sequences affect both genes expression in the same manner suggesting that this will be true for all the genes containing such sequences. Furthermore, most of the Pu27-HS affect cells similarly as Pu27 suggesting that they are interchangeable, and confirmed the binding of the Pu27-HS to the same sequences in the gene promoters. This will be in concordance with different regulatory mechanisms utilized by the Pu27-HS depending most likely on the binding site (in the gene or 5’UTR-mRNA) and the cell type/condition (see further for possible mechanisms in Fig 10). It is however interesting to note that while Pu27 downregulated *c-MYC* and *SOX2* expression (silencing these genes) Pu3+ -which is located in the SOX2 transcribed region- upregulated these two genes expression suggesting different mechanisms of action for Pu27 and Pu3+ to control their respective gene expression at least in this leukemic cell line. It is possible that the difference in gene regulation for Pu27 and Pu3+ depend on the position of their genomic counterpart within their respective gene. Notably, U937 cells were not very sensitive to Pu3+ at day 5 (Fig 2), however by day 6 about 80% growth inhibition was reached (FR observations). We have also observed a significant down-regulation of SOX2 gene expression in U937 exposed to Pu3+ when cultured at higher density (FR observations). These observations suggest that *SOX2* is under a more complex regulatory network most likely dependent on the cell number. It has been suggested that small increase in SOX2 protein can trigger differentiation in mouse embryonic cells [80] it is therefore possible that the increase in expression obtained after Pu27-HS exposure could orient the U937 cells towards differentiation. While we have demonstrated that Pu27 binds to the silencer in c-MYC promoter little is known about G-quadruplex involved in the regulation of SOX2 transcription. Further investigation of G-quadruplex induced regulation of such a key gene as SOX2 would be of the extreme importance for the understanding of cancer and neural development. In addition, the evaluation of the effect each Pu27-HS for the regulation of their respective genes will be informative of the specific role played by these particular G-quadruplex sequences in the cell biology and will likely provide additional possibilities for applications in cancer and stem cell therapy.

We demonstrate in this study that the inhibition of cell proliferation of the U937 cell line by the Pu27-HS was associated with cell cycle arrest at the G1 and S phases suggesting an effect at different regulatory points of the cell cycle. c-MYC is a transcription factor that functions as a transcription regulator for genes in the G1 to S phase transition (E2F1, CDK1…), for genes involved in DNA replication and in the check points (CHK1, CHK2, GADD45…) thus interfering with the cell cycle progression at different level [81]. Notably, it has been shown that c-MYC accelerates the cell entry into G1 phase, the G1 to S transition and the S-phase progression [68–70]. Therefore, a decrease in c-MYC expression would explain the cell cycle arrest where cells will not complete mitosis resulting in inhibition in cell proliferation as observed in cells exposed to Pu27 homologous sequence for 5 days.

Although the exact mechanism by which Pu27 inhibits c-MYC expression is not known, it is likely mediated through direct interaction of the oligonucleotide sequence with its genomic counterpart in c-MYC promoter region, either by intermolecular quadruplex formation or by strand invasion [82]. Our in vitro experiments in which Pu27 (G-rich) was covalently crosslinked to its target sequence clearly indicates that the sequence-specific binding occurs with the C-rich strand of the NHEIII1, via strand invasion. Since Watson-Crick binding of the Pu27 oligonucleotide to the C-rich genomic target would inherently stabilize the G-quadruplex structure of the endogenous Pu27 sequence, the transcription machinery would be repressed (1 in Fig 10). This is very likely the mechanism by which Pu27 oligonucleotide sequences inhibit c-MYC expression. The high sequence similarity of the Pu27-HS suggests that they will preferably bind to the C-rich strand as well. We confirmed that most of the Pu27 family members significantly reduced c-MYC protein expression in leukemic cells. However, this effect was not always the result of a downregulation of c-MYC transcription. Indeed, in some cases the protein expression was reduced even when there was no effect on the c-MYC transcription. This discrepancy could either be result of errors in the transcription of the new mRNA due to the binding of the oligonucleotide sequences to their target NHEIII_1_ in Triplex manner (2 in Fig 10), or of the binding of the oligonucleotides sequences to the 5’UTR-mRNA (3 in Fig 10) as antisense, resulting in both cases in decrease in the translation [83]. At this stage of our investigation, we cannot exclude the possible binding of the exogenous sequences to the G-rich strand of the NHEIII_1_ in vivo to form intermolecular G-quadruplex, stabilizing the G-quadruplex to silence transcription (4 in Fig 10) [9]. More studies will be necessary to extensively investigate the actual binding of these sequences in vivo in order to determine exactly how they work. Nevertheless, at this point of our knowledge and with the evidences presented in this study we have proposed a summary of possible mechanisms for Pu27/Pu27-HS inhibition of c-MYC expression in Fig 10.

It is interesting to speculate about the reason for the existence of these G-quadruplex motifs which contain a similar core of oligonucleotides sequence but are located in different chromosomes. It is possible that quadruplex-forming sequences represent a generalized mechanical feature as suggested by Sen et al. [1, 84] to stabilize the pairing of homologous chromosomes during meiosis or a common mechanism (protein binding, RNA binding…) to regulate transcription of highly expressed genes such as *c-MYC*. The presence of binding sites for different transcription factors common to all the Pu27-HS is in concordance with shared regulatory mechanism. For instance, the presence of MZF1 binding site(s) in all the Pu27 homologous sequences [61] favors a common mechanism especially in regards to the fact that this transcription factor is involved in differentiation of hematopoietic cells [85]. MZF1 may be involved in an early stage of hematopoiesis and plays a role in terminal differentiation–especially of granulocyte lineage [85], it has also been shown to regulate c-MYC expression in lung adenocarcinoma [61]. Furthermore, NHEIII_1_ requirement for *c-MYC* transcription silencing is dependent upon formation of G-quadruplex structures [9, 40, 86]. This 3D folding of the segment of DNA will mask the binding of the TF sites thereby impeding c*-MYC* transcription. In leukemia, c-MYC overexpression may be caused by translocation or amplification implying that the control normally exerted by NHEIII_1_ through stable G-quadruplex structure is abolished, thereby freeing TF binding sites that could promote *c-MYC* expression. The addition of exogenous Pu27 that bind specifically to NHEIII_1_ could stabilize the G-quadruplex either by strand invasion of the complementary C-rich strand (as suggested by the crosslinking experiment describe herein) or by Hoogsteen hydrogen bonds on the G-rich strand [82] occupying MZF1 and/or other TF(s) binding sites to restore the regulatory potential of NHEIII_1_.

Nevertheless, the finding of these almost identical nucleotide sequences located in the promoter region of genes involved in stem cell maintenance such as *c-MYC* and *SOX2*, neural cell differentiation *(SOX2*, *NAV2*, *GRM6)* or near gene involved in senescence such as *BRD7* suggested a shared regulatory mechanism involving sequence specific interactions of either genomic DNA sequences or transcripts of G-quadruplex-forming sequences. Regardless, we believe that the growth inhibitory effects of the Pu27 family of oligonucleotides may provide an opportunity to effectively target c-MYC expression that could be exploited for cancer therapy.

## Supporting Information

S1 Fig (A): Circular dichroism spectra for 4 extra oligosequences from the Pu27 family compared to Pu27. (B): Specificity of four extra Pu27-HS binding to Pu27 target sequence in the c-MYC promoter. (C): Specificity of Pu27 binding to its target sequence, no competition observed by a different G-quadruplex forming oligonucleotide sequences (K-RAS). (D): Specificity of Pu27 binding to its target sequence in the c-MYC promoter, no binding with the G-quadruplex forming oligonucleotides AS1411 or K-RAS.
(PDF)

S1 Table Primers pairs for RT-qPCR analysis: to determine expression of the Pu27 genomic family and target c-MYC and SOX2. (PDF)
