# Supplementary material for: Discovery of a Family of Genomic Sequences Which Interact Specifically with the c-MYC Promoter to Regulate c-MYC Expression
Source: PLoS One. 2016 Aug 23;11(8):e0161588. doi: 10.1371/journal.pone.0161588 (PMC4995011; doi:10.1371/journal.pone.0161588)
Supplement: S1 Fig — (A): Circular dichroism spectra for 4 extra oligosequences from the Pu27 family compared to Pu27. (B): Specificity of four extra Pu27-HS binding to Pu27 target sequence in the c-MYC promoter. (C): Specificity of Pu27 binding to its target sequence, no competition observed by a different G-quadruplex forming oligonucleotide sequences (K-RAS). (D): Specificity of Pu27 binding to its target sequence in the c-MYC promoter, no binding with the G-quadruplex forming oligonucleotides AS1411 or K-RAS. (PDF) [file pone.0161588.s001.pdf]

Supplementary Information

The search for sequence similarity with Pu27 using BLAT on the human GRch37 genome assembly [UCSC Genome Browser (http://genome.ucsc.edu)] identified 13 homologous sequences presenting from 88% to 100% homology with Pu27, localized on different chromosomes. A second search using the updated search engine on GRCh38 revealed 4 more sequences. The study of the last sequences was performed later, after all the data for the 14 sequences were collected therefore using different batch of oligonucleotides and conditions. The data however confirmed the presence of G-quadruplexes as shown in the CD spectra for all sequences comparable to what is obtained with Pu27 (S1 Fig A). The electrophoresis assay (EMSA) shows that the extra sequences of Pu27 family bind to the target sequence in *c-MYC* promoter region and that the binding can be competed by unlabeled Pu27 (S1 Fig B) as observed for the 14 sequences described in the main text (Fig 1E) suggesting that all the sequences similar to Pu27 bind in the same manner to the target sequence. S1 Figure C demonstrates that the binding to the target sequence is specific to the nucleotide sequence as the G-quadruplex forming sequence K-RAS did not displace the binding of Pu27 to *c-MYC* silencer. Furthermore, K-RAS or AS1411 (another well studied G-quadruplex forming sequence) did not bind to Pu27 target sequence in the *c-MYC* promoter (S1 Fig D).

S1 Figures

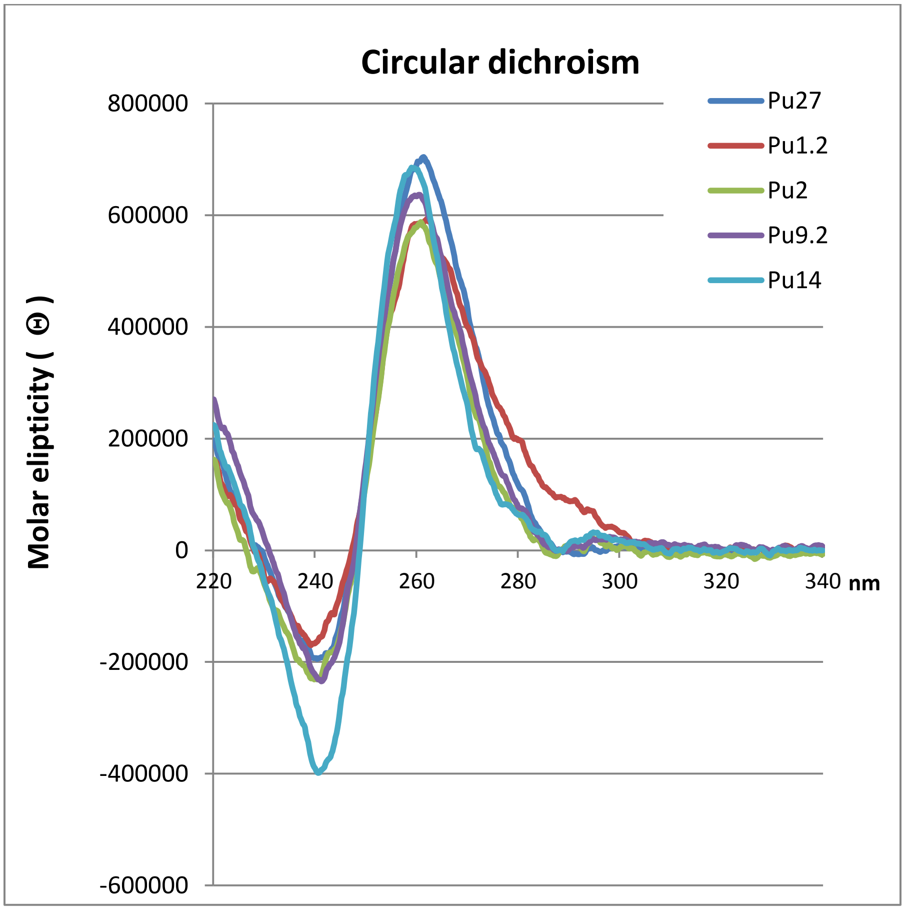

S1 Figure A: Circular dichroism spectra for 4 extra oligosequences from the Pu27 family compared to Pu27. All sequences form parallel G-quadruplex as shown by the presence of a peak at 260nm.

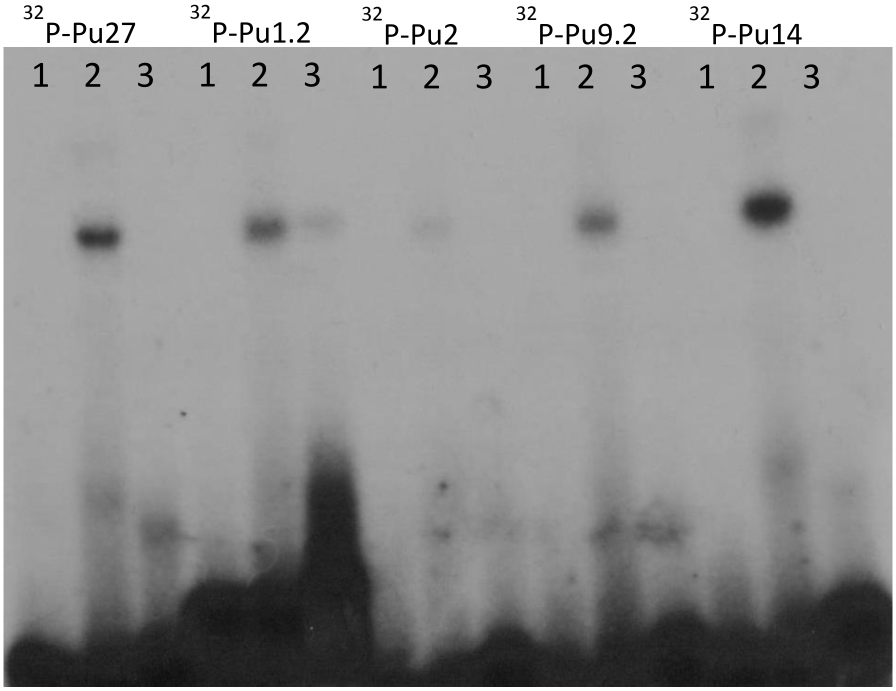

For each sample: Lane#1= oligo ^{32}P-labeled
Lane#2= oligo ^{32}P-labeled + 134bp target
Lane#3= oligo ^{32}P-labeled + 134bp target + 1nM cold Pu27

S1 Figure B: **Specificity of four extra Pu27-HS binding to Pu27 target sequence in the c-MYC promoter**. Photograph of EMSA gel for ^{32}P-labeled-Pu27, ^{32}P-labeled-Pu1.2, ^{32}P-labeled-Pu2, ^{32}P-labeled-Pu9.2 and ^{32}P-labeled-Pu14 run in the presence of a 134bp target sequence containing $NHEIII_1$ of the *c-MYC* promoter +/- 1nM cold Pu27. Kodak image is shown.

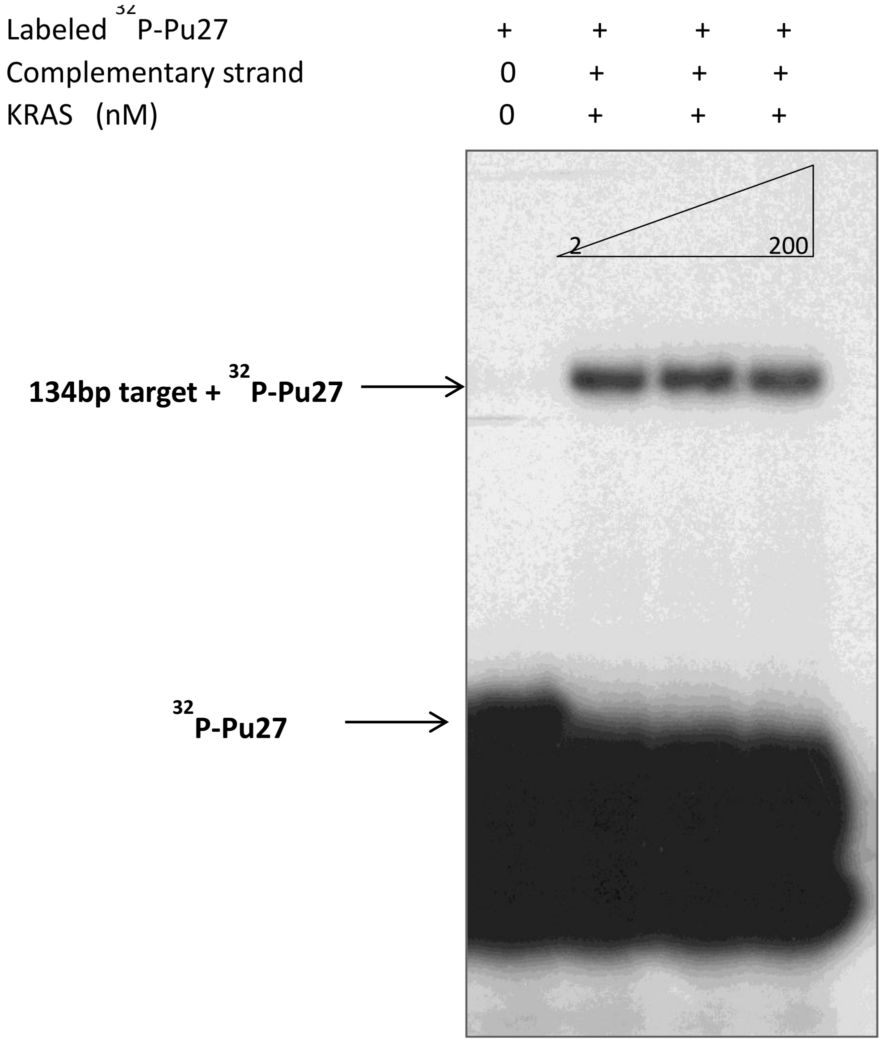

S1 Figure C: **Specificity of Pu27 binding to its target sequence, no competition observed by a different G-quadruplex forming oligonucleotide sequences (K-RAS)**. Photograph of EMSA for ^{32}P-labeled-Pu27 run in the presence of 134bp target sequence containing $NHEIII_1$ of the *c-MYC* promoter +/- 2, 20 and 200nM cold KRAS oligonucleotide sequence, Kodak image is shown.

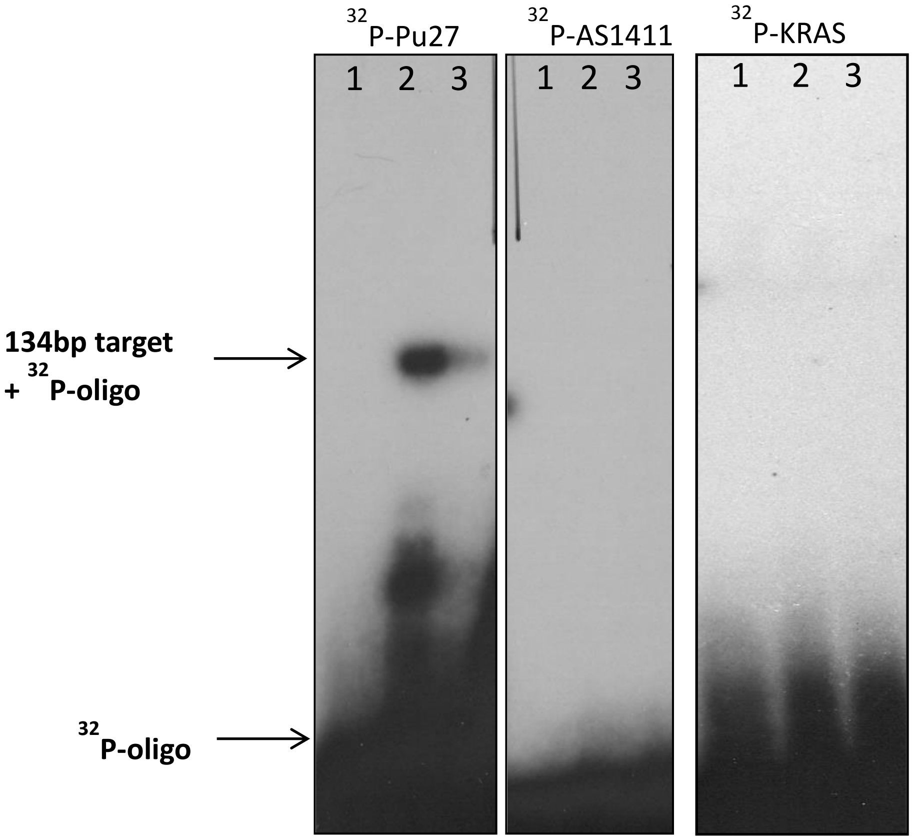

For each sample: Lane#1= oligo ^{32}P-labeled
Lane#2= oligo ^{32}P-labeled + 134bp target
Lane#3= oligo ^{32}P-labeled + 134bp target + 1nM cold Pu27

S1 Figure D: **Specificity of Pu27 binding to its target sequence in the c-MYC promoter, no binding with the G-quadruplex forming oligonucleotides AS1411 or K-RAS**. Photograph of EMSA gel for ^{32}P-labeled-Pu27, ^{32}P-labeled-AS1411, ^{32}P-labeled-KRAS run in the presence of a 134bp target sequence containing NHEIII$_1$ of the *c-MYC* promoter +/- 1nM cold Pu27. Kodak image is shown.
