# Supplementary material for: Discovery of a Family of Genomic Sequences Which Interact Specifically with the c-MYC Promoter to Regulate c-MYC Expression
Source: PLoS One. 2016 Aug 23;11(8):e0161588. doi: 10.1371/journal.pone.0161588 (PMC4995011; doi:10.1371/journal.pone.0161588)
Supplement: S1 Table — (PDF) [file pone.0161588.s002.pdf]

S1 Table: Primers pairs for RT-qPCR analysis: to determine expression of the Pu27 genomic family and target c-MYC and SOX2

Sample ID	Forward	Reverse
**Pu27**	CGGAGATTAGCGAGAGAGGA	TAGGCGCGCGTAGTTAATTC
**Pu 1**	GGTGCAGAGGTCTGGGAGAT	GCTGTGAGCTTCTCCTCACC
**Pu1.2**	AGCTCCTGCCCCCTATAGAA	TCCAGCCTAATCACCAATCC
**Pu2**	TGGGGACTCTGGAGTTCAGT	GCACCAGAGCAGTCTACCG
**Pu3-**	ATGAGCACACATGGGCATAA	TGCATATCATGTGGGTTTGG
**Pu3+**	CGATGCCGACAAGAAAACTT	GCAAACTTCCTGCAAAGCTC
**Pu5**	ACCCAGTCAGACGATTCCAC	GGATGCCCAGAGAAAGTGTG
**Pu7**	CCAGCGTTCCTCAGTCTTGT	ACCTTTGCTAAGCGACTCCA
**Pu9**	TGGTCAAAACCATTCAACAAA	TTAGAGGTGCCCTTCCCTTC
**Pu9.2**	TGGTCAAAACCATTCAACAAA	TTAGAGGTGCCCTTCCCTTC
**Pu10.1**	GGTGGCCCCAAACTGTTAAA	GTACAGGGGTGGCAAAGAAG
**Pu10.2**	AAGGAGAAGAAAGCGCATGT	CTGCAGGAAGAGGTGCAAGT
**Pu11**	AGGAGGGAGGATCCTTTGTT	CTCCGAGTAGCTGGGACTA
**Pu14**	TCCCATTTACATGAAACATCCA	GCAGCCAGCACCACAATTTA
**Pu16**	CATCTTCCAAGGTCAGCACA	GTCCCTGTGGACCCTAACAA
**Pu17**	TCTTGGCATCGTGACTTCAG	ACTGGCCTTTCCAGCTCTTT
**Pu20**	CTCAGCAGAAGGGACAGCAT	AGAGCTAGGAGAGGGGATCG
**PuX**	CACGGAAGTTGTGAGTGGAG	CTCCGCACTCTGCCTCTT
**c-MYC**	CGTCTCCACACATCAGCACAA	TCTTGGCAGCAGGATAGTCCTT
**SOX2**	TACAGCATGTCCTACTCGCAG	GAGGAAGAGGTAACCACAGGG
**GAPDH**	TCAACGACCACTTTGTCAAGCTCA	GCTGGTCCAGGGGTCTTACT
**18S**	GTAACCCGTTGAACCCCATT	CCATCCAATCGGTAGTAGC
